# A Novel Composite Vaccine Combining Inactivated Antigen and IgY Elicits Sustained Humoral Immunity Against FAdV-4 Viruses and PEDV Viruses

**DOI:** 10.3390/v17121569

**Published:** 2025-11-30

**Authors:** Wenming Gao, Zongmei Huang, Lin Liu, Lijie Li, Huimin Huang, Jingrui Liu, Wenwen Zhou, Yapeng Song, Xinsheng Li

**Affiliations:** 1 Department of Microbiology and Immunology, College of Veterinary Medicine, Henan Agricultural University, Zhengzhou 450002, China; 2 College of Veterinary Medicine, Henan University of Animal Husbandry and Economy, Zhengzhou 450002, China

**Keywords:** yolk antibody IgY, immune complex vaccine, fowl adenovirus serotype 4, porcine epidemic diarrhea virus

## Abstract

Vaccination remains the primary strategy for controlling infectious diseases in farm animals. However, current conventional vaccines demonstrate clinical limitations including suboptimal immunogenicity and frequent booster requirements, which compromise disease management efficacy. This study presents an innovative vaccine platform combining yolk immunoglobulin (IgY) with inactivated antigens as co-immunization components. We developed two formulations targeting economically significant pathogens: avian Fowl Adenovirus Serotype 4 (FAdV-4) and Porcine Epidemic Diarrhea Virus (PEDV). For FAdV-4 vaccine evaluation in specific pathogen-free (SPF) chickens, the IgY-antigen complex demonstrated superior immunogenic properties compared to conventional inactivated vaccines. When administered as a single dose at 14 days of age, the experimental formulation elicited significantly stronger humoral responses as measured by both serum neutralization (SN_50_) and ELISA. Notably, this vaccination strategy provided 100% protection against lethal FAdV-4 challenge from 0 h to 20 weeks post-vaccination, with complete absence of clinical disease manifestations. In PEDV assessment using mouse models, the IgY-antigen formulation induced significantly higher antibody titers than inactivated antigen alone at all post-immunization timepoints (*p* < 0.01). Comparative analysis revealed our dual-component platform enhanced both the intensity and rapidity of protective immune responses compared to traditional inactivated vaccines. These findings establish that the IgY-antigen co-immunization strategy represents a promising approach for developing new veterinary vaccines with improved protective efficacy. The platform’s ability to generate robust, rapid-onset immunity while maintaining single-dose effectiveness addresses critical limitations of current vaccine technologies.

## 1. Introduction

Traditional vaccines remain the fundamental tool in preventing and controlling infectious diseases in animal husbandry. Through presenting antigenic components that resemble the pathogens encountered during natural infection, these vaccines elicit host-specific immune responses to establish protective immunity. The main categories comprise inactivated vaccines, live attenuated vaccines, classical subunit vaccines (produced through conventional purification techniques), and toxoid vaccines (targeting toxin-mediated bacterial diseases). Despite their extensive clinical application, traditional vaccines exhibit inherent limitations that constrain their therapeutic potential [1]. A critical evaluation of inactivated vaccines reveals the following advantages and disadvantages. Advantages: (1) Enhanced biosafety. The non-replicative nature of inactivated pathogens eliminates infection risks, particularly critical for vulnerable populations including neonatal and pregnant animals. (2) Improved thermostability. Most formulations maintain immunogenicity under ambient or refrigerated conditions (2–8 °C), with oil-adjuvanted variants demonstrating enhanced temperature tolerance. This characteristic reduces reliance on cold-chain infrastructure, lowering operational expenses and particularly benefiting remote small-scale farming operations. (3) Diagnostic differentiation capacity. When implemented alongside serological monitoring systems (e.g., ELISA-based platforms), these vaccines enable discrimination between natural infection antibodies and vaccine-induced immunity, facilitating epidemiological surveillance in eradication programs. Disadvantages: (1) Restricted immunogenicity profile. Inactivated vaccines primarily induce humoral immunity (antibody-mediated responses) while inadequately stimulating cell-mediated immunity, resulting in suboptimal efficacy against intracellular pathogens. (2) Demanding vaccination protocols. The necessity for multiple booster administrations at specified intervals increases husbandry management complexity and labor requirements. (3) Adjuvant-associated complications. Oil-emulsified formulations frequently induce granulomatous inflammation at injection sites, which may lead to reduced feed conversion efficiency and impaired weight gain in production animals.

Egg yolk antibodies (IgY), a distinct immunoglobulin class unique to oviparous vertebrates (amphibians, avians, and reptiles), undergo selective transport from maternal circulation to yolk sacs during oocyte maturation through receptor-mediated mechanisms [2,3]. While sharing functional similarities with mammalian IgG in antigen recognition, IgY demonstrates structural divergence including heavier chains and additional constant domains [4,5]. This avian immunoglobulin exhibits three key advantages for therapeutic applications: (1) low immunogenicity in mammals, (2) remarkable thermal and pH stability maintaining activity from 4 to 56 °C and pH 4–11, and (3) cost-effective production through natural egg-laying processes [4,5]. These characteristics have enabled widespread applications in passive immunization, showing efficacy against mammalian pathogens such as SARS-CoV-2 [3] and avian viruses including Newcastle disease virus, infectious bursal disease virus (IBDV) [6], avian influenza [6], and poultry reovirus [7]. Mechanistically, IgY exerts dual immunomodulatory functions through antigen-specific Fab regions that neutralize pathogens, coupled with Fc-mediated interactions that enhance phagocytosis and modulate cytokine production [8].

Fowl Adenovirus Serotype 4 (FAdV-4) and Porcine Epidemic Diarrhea Virus (PEDV) continue to pose significant threats to global livestock production. Current vaccine development against FAdV-4 primarily focuses on inactivated vaccines, subunit vaccines, and genetically engineered candidates [8]. Nevertheless, the established protective immunity generally requires 14–21 days post-vaccination, resulting in two critical vulnerability periods: a complete lack of protection during the initial 1–14 days phase and potential immune failure after 21 days.

Akin to FAdV-4 vaccines, conventional PEDV vaccines demonstrate suboptimal protective efficacy when compared to ideal vaccine standards, necessitating the development of novel immunization strategies. To date, no effective vaccine has been developed, due to the continual mutation of the PEDV genome and the low efficacy in inducing mucosal immunity [9,10]. Obtaining sufficient maternal antibody from the colostrum and milk remains the most promising and effective strategy to protect neonatal suckling piglets against PEDV [11,12].

This study developed a dual-pathogen composite vaccine combining IgY with inactivated antigens against FAdV-4 and PEDV. The inactivated virus was used as the aqueous phase and mixed with the oil adjuvant in a certain ratio to prepare a water-in-oil emulsion. Then, the emulsion was combined with the antibody at an appropriate ratio and sheared to prepare a stable complex. The innermost layer of the complex was the antigen, the outermost layer was the antibody, and the middle layer was the oil emulsion. The antigen and antibody were isolated by the oil phase and could not undergo specific binding. We systematically evaluated the vaccine’s immunogenic properties and protective efficacy through in vivo models.

## 2. Materials and Methods

### 2.1. Viruses and Animals

The PEDV CH/HNXX/201606 strain (GenBank accession no. MK124712), maintained in our laboratory, was titrated in Vero cells (CVCC CL28) using a plaque assay (10^5.7^ TCID_50_/mL). The FAdV-4 strain (designated WZ; GenBank accession no. MZ508442) was isolated from hepatic tissue samples of a commercial broiler flock in Henan Province, China. The viral titer of FAdV-4 was quantified by TCID_50_ assay in LMH cell (ATCC CRL-2117) cultures (10^7.8^/0.1 mL). The assay was conducted using standardized endpoint dilution methods [13,14].

Specific pathogen-free (SPF) chickens and BALB/c mice were housed in isolators and individual Ventilated Cages. Fertilized SPF eggs from certified parent flocks were incubated in compliance with AAALAC International guidelines. The embryonation process was conducted under controlled incubation conditions (37.8 °C, 55–60% relative humidity). All animal experimental protocols were ethically reviewed and approved by the Institutional Animal Care and Use Committee of Henan Agricultural University (Approval ID: HNND2019061097).

### 2.2. Preparation of Inactivated Vaccine

The inactivated FAdV-4 and PEDV oil-emulsified vaccines were prepared from their respective parental viral strains. Each viral suspension was inactivated by chemical treatment with 0.1% formalin at 37 °C for 24 h. Residual formaldehyde was subsequently neutralized by adding 1 M glycine. The inactivated virus preparations were emulsified with a commercial oil adjuvant (ISA 71 VG, SEPPIC Chemical Specialities Co., Ltd., Shanghai, China) at an antigen-to-adjuvant ratio of 4:7 (weight-to-weight, *w*/*w*). Negative control vaccines were prepared using identical procedures, with phosphate-buffered saline replacing viral antigens.

### 2.3. Preparation of Egg Yolk Antibodies

To generate anti-FAdV-4 egg yolk antibodies, five specific pathogen-free (SPF) laying hens (aged 160 days) were immunized with an inactivated oil-emulsion antigens. Each hen received three 0.3 mL intramuscular chest injections at 21-day intervals (administered at 160, 181, and 202 days of age), completing the immunization series over a six-week period. The yolk antibodies were subsequently purified through affinity chromatography using a HiTrap™ IgY Purification HP column (Cytiva, Shanghai, China), yielding purified IgY preparations.

Subsequently, we evaluated the neutralizing capacity of these antibodies using a standardized virus neutralization assay [7]. The experimental procedure was conducted as follows: Two 96-well cell culture plates were prepared for parallel processing. One plate received 4 × 10^4.0^ cells per well and was incubated for 24 h to achieve 80% confluency prior to infection. The second plate was preloaded with 100 µL of DME/F-12 medium per well for antibody serial dilution. Antibody titration began with 100 µL of IgY antibody placed in quadruplicate wells (A1, B1, C1, D1), followed by serial two-fold dilutions prepared across 21–22 successive wells. Viral stock of FAdV-4 WZ strain (third passage in F3 cells) was adjusted to 200 TCID_50_/0.1 mL based on the TCID_50_ value established in Experiment 1. Equal volumes (100 µL) of diluted viral solution were combined with each antibody dilution, thoroughly mixed by pipetting, and incubated for neutralization (1 h at 37 °C, 5% CO_2_). The cell monolayer plate was prepared by replacing the existing medium with 100 µL fresh DME/F-12 containing 2% FBS. The antibody-virus complexes were then transferred to corresponding wells of the pre-incubated cell plate. Three essential control groups were established: cell controls (medium only), negative serum controls (non-immune IgY), and virus controls (virus inoculum without antibody). Plates were maintained in a 5% CO_2_ incubator at 37 °C with daily microscopic observation for cytopathic effects (CPE) over 7 days. Neutralization titers were determined by the Reed-Muench method, expressed as the highest antibody dilution achieving 50% protection against CPE development (NT_50_).

### 2.4. Optimization and Preparation of Composite Vaccines

The water-in-oil-in-water (W/O/W) composite vaccine was prepared using a two-step emulsification protocol. Initially, the inactivated viral antigen was homogenized with the oil emulsion adjuvant at a 4:7 (*w*/*w*) ratio following established methodology. Purified IgY antibodies were diluted 20-fold in phosphate-buffered saline (PBS) to prepare the aqueous antibody solution. For formulation optimization, the primary water-in-oil (W/O) vaccine emulsion was then mixed with the specific yolk-derived IgY antibody solution in three distinct volume ratios (W/O vaccine: antibody solution = 3:1, 4:1, 9:1, *v*/*v*). This secondary emulsification was performed by gradually introducing the oil phase into the aqueous antibody solution under controlled low-speed shearing conditions (1100 rpm for 5 min) to ensure proper droplet formation and interface stabilization.

Vaccine stability was evaluated through centrifugation testing (3000 rpm × 15 min) with formulations demonstrating ≤5% (*v*/*v*) aqueous phase precipitation considered acceptable for physical stability. For comprehensive characterization, vaccine formulations underwent dynamic light scattering (DLS) analysis (25 °C, 0.1 mg/mL in PBS) to determine particle size distribution and zeta potential parameters critical for assessing colloidal stability and surface charge properties.

The composite vaccine containing inactivated virus and specific yolk immunoglobulin (IgY) was prepared using a two-step emulsification method. Each 0.5 mL dose consisted of 10^6^ TCID_50_ inactivated virus mixed with IgY at a 9:1 (*v*/*v*) ratio (virus suspension/IgY solution). During formulation, antigen–antibody complexes were formed via specific binding between viral surface proteins and therapeutic antibodies in IgY. The emulsion was vortex-mixed at moderate speed (500 rpm) for 5 min to ensure component homogeneity while maintaining complex stability. The final vaccine formulation was stored at 4–8 °C prior to immunization.

### 2.5. FAdV-4 Vaccination and Challenge

Specific pathogen-free (SPF) chickens were housed in isolators with ad libitum access to water and standard poultry feed. The study cohorts (*n* = 65/group) comprised three randomly assigned treatment groups: Group A received 0.5 mL of FAdV-4 immune complex formulation (containing FAdV-4 antigen–antibody complexes) via pectoral muscle injection at 14 days post-hatch; Group B was administered 0.5 mL/bird of inactivated FAdV-4 vaccine formulated in oil emulsion adjuvant through the same intramuscular route; Group C served as the emulsion control with equivalent-volume injections of PBS-formulated oil emulsion.

To assess vaccination safety, longitudinal growth monitoring was implemented with weekly recording of body weight (g). Blood collection protocols occurred weekly from vaccination week 1 through trial termination. Serum samples were centrifuged (3000× *g*, 10 min) and stored at −80 °C pending FAdV-4 neutralizing antibody quantification using virus neutralization assays and ELISA tests.

To assess vaccine-induced protection, five specific pathogen-free (SPF) chickens per group were challenged intramuscularly with the FAdV-4 WZ strain (2 × 10^6^ TCID_50_/bird) at designated timepoints after immunization. Challenge timepoints were strategically selected to evaluate both immediate and prolonged immunity: immediately post-vaccination (0 h), 12 h, 24 h, and then at weekly intervals spanning weeks 1 through 20 (1, 2, 3, 4, 5, 8, 12, 16, and 20 weeks). Following challenge, all subjects were observed twice daily for clinical manifestations (including lethargy, anorexia, and neurological signs) and mortality outcomes. To ensure diagnostic accuracy, mortalities underwent immediate postmortem examination (<1 h after death) for macroscopic lesion evaluation. Surviving chickens were maintained under observation for a 14-day period to monitor potential disease progression before being humanely euthanized for comprehensive histopathological analysis.

For parallel reproductive assessment, fertilized eggs were collected daily from immunized breeders (hen/rooster ratio = 4:1) starting at 19 weeks on a daily basis. The collected eggs were incubated to produce chicks for vertical transmission studies. At 2 weeks of age, the offspring were challenged via intramuscular administration of the FAdV-4 WZ strain (2 × 10^6^ TCID_50_/bird). Observation and necropsy protocols were the same as those described for the parent stock.

### 2.6. PEDV Vaccination and Evaluation

A murine model was utilized to evaluate PEDV vaccine efficacy. Thirty 6-week-old BALB/c mice were randomly allocated into three experimental groups (*n* = 10/group): the water-in-oil (W/O) emulsion vaccine group, water-in-oil-in-water (W/O/W) emulsion vaccine group, and PBS-formulated oil emulsion group. Subcutaneous immunization was performed in the dorsal flank region with 0.3 mL of respective vaccine formulations. The immunization protocol consisted of prime vaccination on day 0 followed by two booster doses at 14-day intervals (week 2 and week 4 post-prime), with identical injection parameters maintained throughout. Serial blood samples (≈200 μL) were collected via tail vein puncture at seven timepoints: pre-immunization (day 0) and weekly intervals from weeks 1 to 6 post-immunization. Serum fractions were obtained through centrifugation at 3000× *g* for 15 min, aliquoted into sterile cryovials, and preserved at −80 °C pending serological analysis.

### 2.7. ELISA Test

Serum samples collected from chickens were initially evaluated for anti-FAdV-4 and anti-PEDV antibody levels using enzyme-linked immunosorbent assay (ELISA) according to standardized protocols. The procedure was conducted as follows: Purified FAdV-4 or PEDV antigen (1.75 μg/well) was immobilized onto 96-well plates through overnight incubation at 4 °C. After removing unbound antigens, the plates were blocked with 5% skimmed milk powder in PBST (phosphate-buffered saline with 0.05% Tween 20) for 2 h at 37 °C. Subsequent to blocking solution removal, three sequential washes with PBST were performed using an automated plate washer. Serum samples from experimental groups were diluted 1:1000 in sample dilution buffer and incubated in antigen-coated wells for 1 h at 37 °C. Following primary antibody incubation and three additional washing cycles, horseradish peroxidase (HRP)-conjugated rabbit anti-chicken or goat anti-mouse IgG (Proteintech, Wuhan, China) was added at a 1:7000 dilution in blocking buffer. The secondary antibody incubation proceeded for 1 h at 37 °C with constant agitation. After five final PBST washes, 100 μL of 3,3′,5,5′-tetramethylbenzidine (TMB) substrate (Solarbio, Beijing, China) was dispensed into each well, and the colorimetric reaction was allowed to develop for 10 min in darkness. Reaction termination was achieved by adding 50 μL of 2 M sulfuric acid per well. Optical density measurements were obtained at 450 nm using a SpectraMax M5 microplate reader (Thermo Fisher, Shanghai, China). To maintain experimental validity, inactivated FAdV-4/PEDV-positive serum served as positive controls while healthy serum (never exposed to either virus) functioned as negative controls. All samples and controls were analyzed in triplicate wells across three independent assay plates.

### 2.8. Neutralization Assay

Serum samples from each group were heat-inactivated at 56 °C for 30 min and subjected to two-fold serial dilutions, starting with an initial dilution of 1:4 (*v*/*v*). These diluted serum samples were mixed with 100 TCID_50_ per well of virus in equal volumes (1:1 ratio, *v*/*v*) and incubated at 37 °C under 5% CO_2_ for 1 h. The resulting virus-serum mixtures were then inoculated onto confluent cell monolayers in 96-well plates. Following a 7-day incubation under standard cell culture conditions (37 °C, 5% CO_2_, 95% humidity), cells were fixed with crystal violet and microscopically evaluated for cytopathic effects (CPE).

Neutralization activity was determined by comparison with parallel controls: virus-only controls (to verify viral infectivity) and cell-only controls (to assess background cell viability). A serum sample was considered neutralizing if it demonstrated ≥50% reduction in CPE relative to virus-only controls.

### 2.9. Analysis of Viral Shedding

Following experimental challenge with FAdV-4, cloacal swab samples were collected from both immunized and control chickens. The swab samples were suspended in PBS and stored at 4 °C for 1 h. Subsequent centrifugation at 8000× *g* for 10 min separated the liquid components, after which the supernatant was carefully collected. Nucleic acid extraction was then performed using a commercial DNA extraction kit, strictly adhering to the manufacturer’s instructions. To assess FAdV-4 infection dynamics, we implemented quantitative PCR (qPCR) for precise copy number determination [15]. Viral load quantification employed the 2−ΔΔCt method with β-actin as the endogenous reference control.

### 2.10. Histopathological Studies

Under experimental challenge conditions, heart, liver, and kidney samples were collected from chickens for histopathological evaluation. Fresh tissue specimens underwent immediate fixation in 10% neutral buffered formalin maintained at 25 ± 2 °C for 16–18 h. This fixation protocol ensured complete tissue preservation for microscopic analysis. The fixed specimens were then processed through a conventional alcohol dehydration series (70–100% ethanol) followed by paraffin embedding using standard histological protocols. Tissue sections of 5 μm thickness were prepared using a rotary microtome. All sections were stained with hematoxylin and eosin following established histological protocols, with blinded evaluation performed by two independent pathologists using an Olympus BX53 light microscope equipped with a DP27 digital camera system(Olympus, Nagano Prefecture, Japan). Microscopic observations were recorded at 200× and 400× magnifications for detailed cellular assessment.

### 2.11. Statistical Analysis

All data were statistically analyzed using GraphPad Prism 9 and expressed as “mean ± standard deviation”. For survival curves, significance was determined using the log-rank (Mantel–Cox) test. The data were considered significantly different when *p* < 0.05 between different groups in EXCEL version 14.0 software.

## 3. Results

### 3.1. Preparation and Analysis of FAdV-4 Composite Vaccine

**Figure 1:**
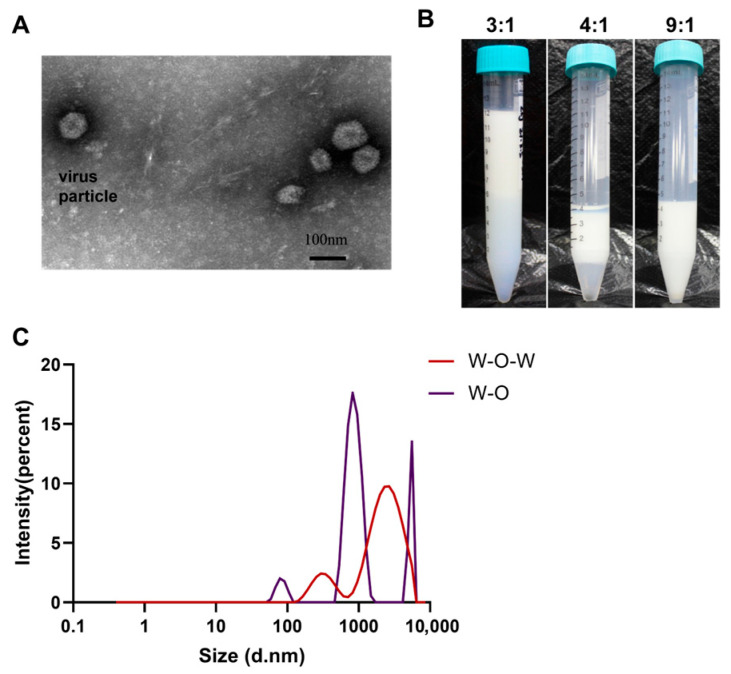
Characterization of virus particles and stability test results of the FAdV-4 immune complex. (**A**) Virus particles under an electron microscope. The virus particles were negatively stained and the images were captured by Transmission Electron Microscopy (TEM) at 40,000× magnification. The diameter of the particles detected was about 80 nm. (**B**) Mix of inactivated viruses with antibodies. The inactivated FAdV-4 WZ strain was mixed with specific yolk antibodies in three different ratios (3:1, 4:1, 9:1, respectively). (**C**) Dynamic light scattering data for combination vaccine. The hydrodynamic molecular radius of different conditions by DLS are 0.864 μm and 2.655 μm, respectively.

We formulated the composite Fowl Adenovirus serotype 4 (FAdV-4) vaccine containing 10^6^ TCID_50_/0.5 mL of FAdV-4 WZ strain (Figure 1A) combined with 5 × 10^3.0^ PD_50_ of specific yolk immunoglobulins. Stability testing of the water-in-oil-in-water (W/O/W) emulsion was performed through centrifugation at 3000 rpm for 15 min. The optimized preparation (9:1 emulsion-to-aqueous phase ratio, *v*/*v*) demonstrated excellent physical stability after centrifugation, showing two distinct properties: (1) limited oil phase sedimentation (<5% total volume) accompanied by the absence of observable phase separation, and (2) immediate recovery of homogenous dispersion with typical milky-white turbidity following gentle shaking (Figure 1B). Dynamic light scattering measurements indicated a marked increase in particle diameter for the W/O/W emulsion compared to standard inactivated vaccine formulations, expanding from 0.864 ± 0.12 μm to 2.655 ± 0.31 μm (Figure 1C). Through comprehensive physicochemical evaluation, the optimal preparation protocol was established as: water-to-oil phase ratio of 4:7 (*w*/*w*), combined with 9:1 (*v*/*v*) emulsion-to-aqueous phase ratio.

### 3.2. Body Weight Changes After FAdV-4 Composite Vaccine Immunization

**Figure 2:**
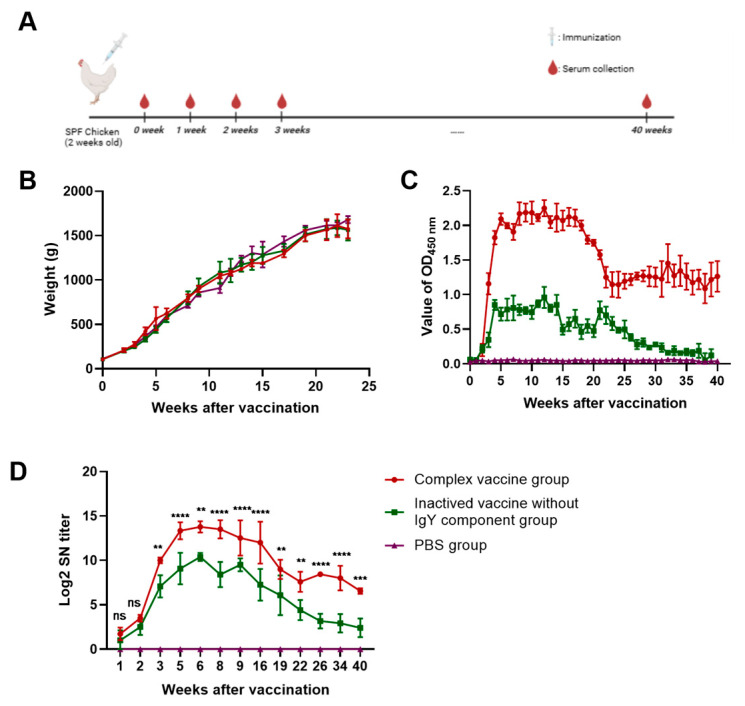
Growth curve and anti-FAdV-4 antibody titer of immunized SPF chickens. (**A**) 2-week-old SPF chickens were immunized with complex vaccine, inactived vaccine without the IgY component, and PBS, respectively. Serum samples were collected weekly until 40 weeks after vaccination. “……” means samples were collected weekly. (**B**) The weight gain of chickens after immunization was monitored and recorded. (**C**) The level of FAdV-4 specific antibodies in chicken serum samples post-immunization was determined by ELISA. (**D**) The measurement of neutralizing antibodies. The neutralizing titer (NT) of sera from the inoculated chickens were tested from 1 to 40 weeks pv. Data are shown as the mean ± SD. Statistical significance was determined using one-way ANOVA. ns, no significance; **, *p* < 0.01; ***, *p* < 0.001; ****, *p* < 0.0001.

The schedule for vaccination and serum sampling was shown in Figure 2A. To evaluate the safety profile of the composite vaccine candidate, we systematically monitored growth metrics in chickens throughout the 14-day post-immunization observation period. Vaccinated chickens exhibited normal activity, feeding patterns, and fecal morphology without observable adverse effects. The vaccine candidate demonstrated adequate systemic absorption, with gross pathological examinations showing no visible tissue alterations in internal organs. Longitudinal analysis revealed comparable growth trajectories between vaccinated and control chickens. Statistical analysis demonstrated no significant difference (*p* > 0.05) in terminal body weight at 23 weeks post-immunization (Figure 2B).

### 3.3. Antibody Response of FAdV-4 Composite Vaccine

The humoral immune response against FAdV-4 in chickens was assessed longitudinally through serum analysis using indirect ELISA. Pre-immunization screening confirmed that all subjects were seronegative for maternal antibodies against FAdV-4. Group A consistently demonstrated significantly higher ELISA antibody titers than Group B during the observation period, accompanied by a markedly slower rate of decline (Figure 2C). Both groups exhibited rapid seroconversion beginning at 2 weeks post-vaccination (pv), reaching peak antibody levels by week 5 pv. A gradual decrease in antibody titers was observed from week 16 to 40 pv in both vaccination groups. These results indicate that the FAdV-4 immune complex formulation induced sustained antibody responses maintaining clinically protective titers for more than 40 weeks pv, demonstrating superior longevity of humoral immunity compared to the inactivated vaccine formulation.

Neutralization assays were performed to evaluate the functional protective efficacy of vaccine-induced antibodies. Comparative analysis revealed that the FAdV-4 immune complex induced significantly higher geometric mean titers (GMT) of neutralizing antibodies (NAbs) than the inactivated vaccine formulation (Figure 2D). Both vaccinated groups demonstrated peak NAb responses at 6 weeks post-vaccination (wpv.), whereas the control group sera exhibited no detectable neutralizing activity. Notably, the two vaccine formulations maintained high-titer NAb responses from 5 to 16 wpv. Subsequent longitudinal analysis showed persistent seropositivity continuing through the final observation timepoint at 40 wpv for the FAdV-4 immune complex group, while equivalent data beyond 16 wpv for the inactivated vaccine require clarification in subsequent studies. The temporal pattern of GMT elevation combined with neutralizing capacity measurements demonstrates that the FAdV-4 immune complex formulation enhances immunogenicity relative to the inactivated vaccine candidate.

### 3.4. The FAdV-4 Composite Vaccine Protects the Host Against Viral Challenge

**Figure 3:**
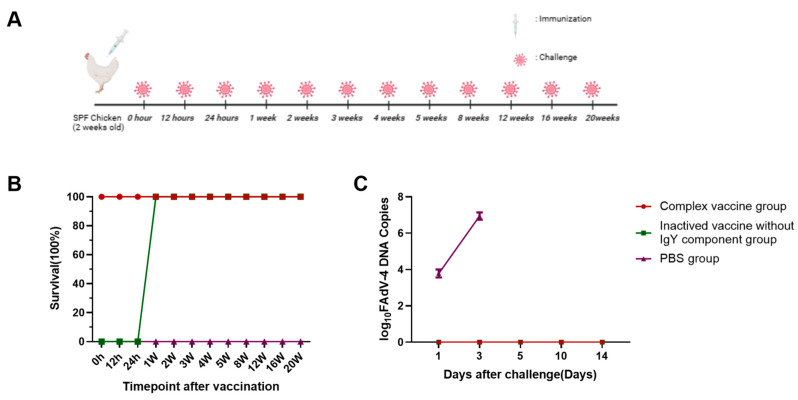
Survival ratios and viral shedding in swab samples of chickens after challenge. (**A**) 2-week-old chickens were immunized with complex vaccine, inactived vaccine without the IgY component, and PBS, respectively, and challenged intramuscularly with FAdV WZ strain (2 × 10^6^ TCID_50_/bird) at 0 h, 12 h, 24 h, 1 weeks, 2 weeks, 3 weeks, 4 weeks, 5 weeks, 8 weeks, 12 weeks, 16 weeks, and 20 weeks after immunization. (**B**) The survival ratio was observed in each group after virus challenge. (**C**) Viral load and shedding kinetics in challenged chickens.

The schedule for vaccination and challenges is shown in Figure 3A. The protective efficacy against the FAdV-4 WZ strain was evaluated at multiple timepoints post-challenge, with daily monitoring of clinical signs and mortality. Chickens in group A maintained complete protection (100%) from 0 h to 20 weeks post-immunization, showing neither clinical symptoms nor mortality throughout the observation period (Figure 3B). Postmortem examinations revealed no vaccine-related pathological lesions in this group. Group B exhibited transient vulnerability during the initial 24 h post-vaccination, showing 0% protection in this early phase. However, complete protection (100%) was established by 1 week post-vaccination. Both vaccinated groups sustained full protection without disease manifestations from 1 wpv through the study endpoint at 20 wpv. Notably, all control chickens (group C) developed fatal infections, with 100% mortality occurring within 3 days post-infection (dpi). These findings demonstrate that the FAdV-4 immune composite vaccine induces durable immunity with immediate onset of complete protection, contrasting with conventional inactivated vaccines that require 7 days to develop full efficacy.

Viral shedding patterns were confirmed through qPCR analysis of oropharyngeal and cloacal swabs collected at scheduled intervals (Figure 3C). No detectable viral DNA was observed in groups A and B from 1 wpv through the 20-week study period. In contrast, control animals (group C) exhibited high viral loads as early as 1 day post-challenge. This virological evidence aligns with clinical protection data, confirming that the immune composite vaccine establishes sterilizing immunity immediately after administration. The complete absence of viral shedding in vaccinated groups from the first week post-immunization further demonstrates the vaccine’s capacity to block both clinical disease and viral transmission.

### 3.5. Histopathology of FAdV-4 Composite Vaccine

**Figure 4:**
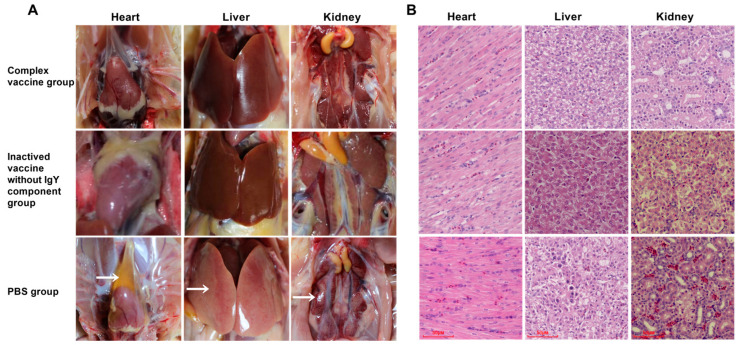
Autopsy results and H&E staining (HC) observation of tissues after virus challenge. (**A**) Pathological analysis of tissues after virus challenge. Hydropericardium Syndrome, inclusion body hepatitis, and mottled kidney are indicated by arrows in the PBS group. (**B**) H&E observation of tissues after virus challenge. Normal histopathology can be observed in the PBS group. The heart, liver, and kidney tissues of chickens from the immunized group showed no histopathological changes. (H&E stain, original magnification 400×, scale bar = 50 μm).

The post-challenge necropsy findings for each experimental group are presented in Figure 4A. Chickens in the immunized groups exhibited normal gross pathology with no visible tissue damage. In contrast, control animals displayed pathological lesions characteristic of FAdV-4 infection, including pericardial accumulation of yellowish exudate, hepatic degeneration with necrotic foci, and severe renal enlargement with marked hemorrhage. Further histopathological analysis of infected tissues (heart, liver, and kidney) revealed distinct pathological changes (Figure 4B). Liver sections showed significant lipid vacuolization and numerous intranuclear inclusion bodies. Renal tissue exhibited extensive lymphocytic infiltration and vascular congestion, while cardiac sections displayed interstitial edema and pronounced vascular engorgement.

Viral shedding dynamics were assessed at different intervals post-challenge (Figure 3B). No viral shedding was detected in Groups A (immune complex vaccine) and B (inactivated vaccine without the IgY component) during the 20-week observation period. Group C (unvaccinated controls) demonstrated detectable viral excretion within 24 h post-challenge. These results align with vaccine efficacy data: the immune complex vaccine provided immediate protection upon administration, whereas the inactivated vaccine without the IgY component required a 7-day induction period to establish complete protective efficacy.

### 3.6. Transmission of Maternal Antibodies

**Figure 5:**
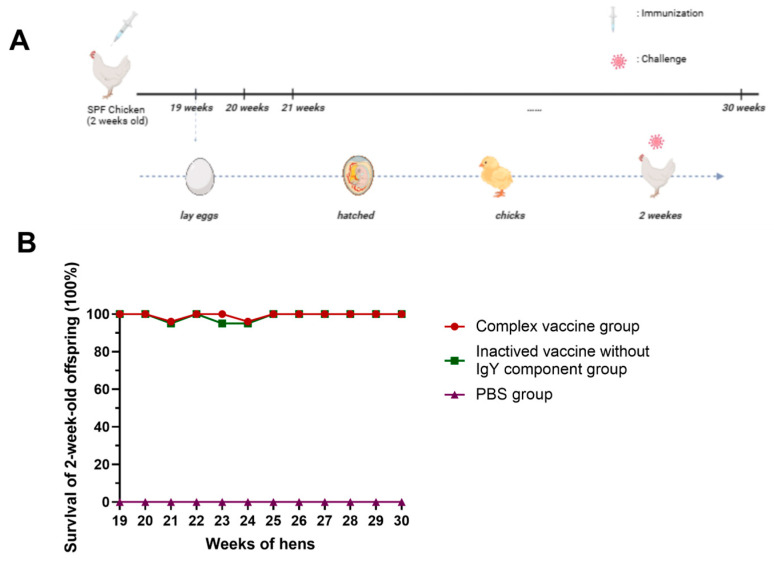
Transmission of protective efficacy of immune complex from breeders to offspring after challenging with FAdV-4. (**A**) 2-week-old SPF chickens were immunized with complex vaccine, inactived vaccine without the IgY component, and PBS, respectively. They were raised to lay eggs at 19-weeks old. The harvested embryos were incubated and raised to 2-weeks old, then challenged intramuscularly with FAdV WZ strain (2 × 10^6^ TCID_50_/bird). “……” means samples were collected weekly. (**B**) Fertilized eggs were collected and incubated from 19-weeks old. The offspring from hens were challenged by intramuscular injection with 2 × 10^6^ TCID_50_/bird of FAdV-4 WZ strain at 2 weeks of age.

The schedule for vaccination and hatching the offspring was shown in Figure 5A. We conducted a challenge study to evaluate the efficacy of maternally transferred antibodies from vaccinated hens in protecting offspring against FAdV-4 infection (Figure 5B). Fertilized eggs collected from 19-week-old to 30-week-old breeder hens were incubated, and twelve independent batches of 2-week-old progeny were intramuscularly challenged with 2 × 10^6^ TCID_50_/bird of the FAdV-4 WZ strain.

Remarkably, the FAdV-4 immune composite vaccine maintained 95–100% protective efficacy in 2-week-old progeny even when derived from hens immunized at 19-week-old to 30-week-old egg collection. In contrast, the PBS-treated control offspring exhibited no protection (0% survival). These results demonstrate that the immune complex vaccine-induced maternal antibodies can provide effective passive immunity against FAdV-4-associated mortality in offspring.

### 3.7. Immune Efficacy of the PEDV Composite Vaccine

**Figure 6:**
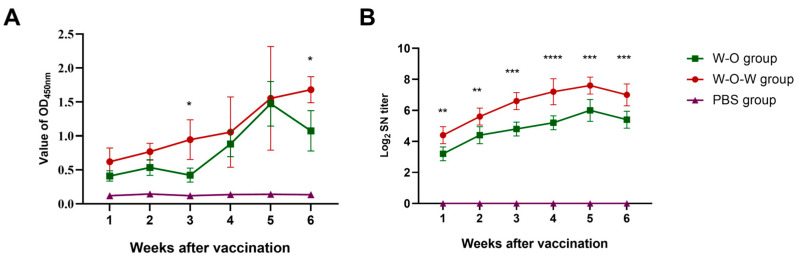
Specific antibody titer of serum against PEDV from BALB/c mice. (**A**) Antibody titer was measured by PEDV antigen ELISA. (**B**) The measurement of neutralizing antibodies. Data are shown as the mean ± standard error. Statistical significance was determined using one-way ANOVA. *, *p* < 0.05; **, *p* < 0.01; ***, *p* < 0.001; ****, *p* < 0.0001.

The immunogenicity of the PEDV composite vaccine was further characterized. Compared to traditional water-in-oil (W/O) adjuvants, the water-in-oil-in-water (W/O/W) complex emulsion adjuvant elicited stronger immune responses in BALB/c mice models. Specifically, the W/O/W formulation induced significantly higher seroconversion and demonstrated extended antibody durability (Figure 6A). The PEDV composite vaccine also induced significantly higher geometric mean titers (GMT) of neutralizing antibodies (NAbs) than the inactivated vaccine formulation (Figure 6B). A significant difference was observed between the W/O/W group and the W/O group. These results indicate that IgY enhances the immune capacity of the inactivated PEDV vaccine.

## 4. Discussion

IgY antibodies have proven to be safe and effective in clinical applications as pathogen-specific biotherapeutics, functioning through high-affinity target binding for microbial neutralization [3,4,5,6]. After oral administration, these antibodies are efficiently absorbed into the gastrointestinal tract and transported to the bloodstream through enteric circulation. Our findings demonstrate that passive immunization with IgY provides temporary but critical immunoprotective coverage during the maturation phase of vaccine-induced active immunity. The established dual-phase protection model involves multiple synergistic mechanisms: target-binding antibodies exert their protective effects through steric hindrance of viral-receptor interaction, pathogen-agglutination precipitation, opsonophagocytic enhancement, and complement-mediated virolysis—working cooperatively to establish robust antiviral defenses [16,17,18].

First identified in China during the 2015 outbreaks, FAdV-4-associated inclusion body hepatitis with pericardial effusion syndrome has prompted the development of immunization-based control strategies [13]. While vaccination remains the cornerstone for disease prevention, emerging evidence suggests a critical 3-week vulnerability window exists between vaccine administration and establishment of protective immunity in poultry flocks. Developing vaccines that provide immediate immunoprotection while maintaining long-term efficacy has therefore become a priority for sustainable avian adenovirus control.

This study developed an innovative active-passive immunization strategy combining formalin-inactivated FAdV-4 with hyperimmune IgY against FAdV-4. Experimental validation in specific-pathogen-free (SPF) Leghorn chicks demonstrated that the immune complex vaccine achieved 100% clinical protection with complete viral clearance by post-challenge from 0 h to 20 weeks, as confirmed through qPCR and histopathological analysis.

Compared to standalone inactivated vaccines, our immune complex formulation demonstrates accelerated initiation, augmented intensity, and extended duration of protective immunity. This superior efficacy is consistent with established mechanisms whereby immune composite vaccines alter the rate or pattern of viral replication while preserving antigen immunogenicity [19]. The water-oil-water (W/O/W) emulsion, engineered through phase fractionation emulsification, achieved optimal physicochemical stability. This delivery system exhibited exceptional formulation homogeneity and enhanced adjuvant properties that synergistically potentiate rather than compromise native immune functions. In the immune complex, the antibody and antigen were physically isolated by the oil phase, and the antigen surface did not be masked by antibody; therefore, they would not undergo specific binding. The antibody and antigen were absorbed or recognized, respectively, at different times, and they play an important role in passive and active immunity. When the immune complex was injected into the animal body, the outer layer of the antibody aqueous phase, which contains a substantial molar excess of IgY antibodies, could be released into the bloodstream quickly, providing immediate passive immunity. However, the IgY antibody has a half-life of approximately 4.1 days [20], As it was absorbed gradually, the internal water-in-oil emulsion began to stimulate the immune system. The antigen aqueous phase was recognized by the immune cells, generating active immunity, and protective antibodies began to appear within 10–14 days. So, this immune complex can facilitate a rapid immune response, and the antigen surface could not be masked by preformed antibodies on the immunogenicity of the co-administered antigen.

The vertical transmission of maternal immunity from vaccinated breeders to progeny represents a critical advantage in FAdV-4 management [8,21]. As demonstrated in previous studies [22,23], vaccine efficacy shows strong correlation with transovarian antibody transfer levels, where maternal antibody titers serve as key determinants of broiler protection. Notably, our immune complex formulation achieved sustained high-level protective efficacy in progeny through 30 weeks post-vaccination while maintaining excellent safety profiles in vaccinated breeders, with histopathological analysis confirming absence of tissue damage. Compared to conventional inactivated vaccines, this dual-action formulation (combining immune complex formation with enhanced antigen presentation) demonstrated accelerated induction of protective immunity through antibody-mediated antigen processing [1,7]. The enhanced immunogenicity likely results from improved phagocytosis and intracellular processing of high-affinity immune complexes by professional antigen-presenting cells [24].

To investigate the potential application of egg yolk antibodies in composite vaccine development for non-avian species, we formulated a combined PEDV-IgY vaccine. IgY from immunized chickens has also been shown to be safe and effective against PEDV in newborn piglets [25]. In previous studies, due to the incomplete development of the digestive system in newborn piglets, IgY taken through the digestive tract can quickly and effectively penetrate the intestinal wall of newborn piglets, maintain long-term activity in the intestine, and have a significant protective effect on piglets. The IgY produced by purified recombinant PEDV S1 protein immunized hens can also protect piglets against PEDV infection [26]. Throughout, IgY against PEDV may have been an alternative method of supplementing prophylactic measures like colostral antibodies from sows. In this study, we formed a novel composite vaccine by combining inactivated PEDV antigen with IgY, which triggered sustained humoral immunity and represents a novel application of IgY in PEDV prevention and control. These findings suggest that the egg yolk antibody composite vaccine exhibits potential applicability in non-avian species.

Functionally analogous to mammalian IgG, avian IgY demonstrates inherent immunomodulatory properties. From an economic perspective, the utilization of egg yolk antibodies presents a more cost-effective alternative to IgG. Therefore, the IgY immune complex vaccine represents a promising candidate with significant commercial potential.

## 5. Conclusions

We developed water-in-oil-in-water (W/O/W) emulsion immune complexes containing precisely formulated ratios of rapid-acting antibodies and sustained-release antigens for controlled in vivo release. The monovalent immune composite vaccine demonstrated 100% protective efficacy in challenged birds, sustaining protection from immediately after immunization (0 min) through 20 weeks post-vaccination. This formulation effectively bridged the immunity gap between initial vaccination and adaptive immune response maturation. Notably, protective immunity against FAdV-4 infection was vertically transmitted through maternal antibodies, with neutralizing antibody titers in progeny maintained at ≥6.2 log2 for up to 3 weeks post-hatching. These findings demonstrate that this complex formulation represents a promising vaccine candidate. Furthermore, it provides a novel strategy for the global control of Hydropericardium Syndrome (HHS) outbreaks through combined immediate and sustained protection mechanisms.

## Data Availability

The original contributions presented in this study are included in the article. Further inquiries can be directed to the corresponding author(s).
